# Treatment and life goals among veterans with Gulf War illness

**DOI:** 10.1371/journal.pone.0295168

**Published:** 2023-11-30

**Authors:** Nicole Sullivan, Hannah Schorpp, Sarah Crosky, Scott Thien, Drew A. Helmer, David R. Litke, Wilfred R. Pigeon, Karen S. Quigley, Lisa M. McAndrew

**Affiliations:** 1 War Related Illness and Injury Study Center, Veterans Affairs New Jersey Health Care System, East Orange, NJ, United States of America; 2 Department of Psychology, William Paterson University, Wayne, NJ, United States of America; 3 Department of Educational and Counseling Psychology, University at Albany, Albany, NY, United States of America; 4 Center for Innovations in Quality, Effectiveness, and Safety, Michael E. DeBakey Veterans Affairs Medical Center, Houston, TX, United States of America; 5 Department of Medicine, Baylor College of Medicine, Houston, TX, United States of America; 6 Department of Rehabilitation Medicine, New York University Grossman School of Medicine, New York, NY, United States of America; 7 Center of Excellence for Suicide Prevention, Canandaigua VA Medical Center, Canandaigua, NY, United States of America; 8 Department of Psychiatry, University of Rochester Medical Center, Rochester, NY, United States of America; 9 Department of Psychology, Northeastern University, Boston, MA, United States of America; Keele University School of Medicine, UNITED KINGDOM

## Abstract

Medically unexplained syndromes (MUS), also termed persistent physical symptoms, are both prevalent and disabling. Yet treatments for MUS are marked by high rates of patient dissatisfaction, as well as disagreement between patients and providers on the management of persistent physical symptoms. A better understanding of patient-generated goals could increase collaborative goal setting and promote person-centered care, a critical component of MUS treatment; yet research in this area is lacking. This paper aimed to develop a typology of treatment and life goals among Gulf War veterans with a medically unexplained syndrome (Gulf War Illness). We examined participants’ responses to open-ended questions about treatment and life goals using Braun and Clarke’s thematic analysis methodology. Results showed that treatment goals could be categorized into four overarching themes: 1) Get better/healthier, 2) Improve quality of life, 3) Improve or seek additional treatment, and 4) Don’t know/Don’t have any. Life goals were categorized into six overarching themes: 1) Live a fulfilling life, 2) Live a happy life, 3) Live a healthy life, 4) Be productive/financially successful, 5) Manage GWI, and 6) Don’t know/Don’t have any. Treatment goals were largely focused on getting better/healthier (e.g., improving symptoms), whereas life goals focused on living a fulfilling life. Implications for the treatment of Gulf War Illness and patient-provider communication are discussed.

ClinicalTrials.gov Identifier: NCT02161133.

## Introduction

Medically unexplained syndromes (MUS), also termed persistent physical symptoms, are illnesses that manifest as clusters of physical symptoms without a known biological cause, such as fibromyalgia and chronic fatigue syndrome. These conditions are not only prevalent (affecting up to 34% of patients within primary care settings), but also chronic and disabling [1]. Certain subpopulations, such as Gulf War veterans (former military service members deployed to the Persian Gulf region in 1990–1991), have particularly high rates and severity of persistent physical symptoms known as Gulf War Illness (GWI) [2]. GWI consists of the presence of chronic, medically unexplained symptoms in at least 3 of 6 symptom clusters, which include pain, fatigue, neurocognitive, respiratory, skin, and gastrointestinal domains [3].

While clinical practice guidelines recommend non-pharmaceutical, behavioral approaches to GWI management [4, 5], it is well-documented that patients with MUS (including Gulf War veterans) receive excessive medical testing and pharmacological treatment, much of which is ineffective, inefficient, and costly [6–9]. The persistence of this disease-driven approach to MUS care may be due, at least in part, to providers believing that patients with MUS want medical interventions and would reject behavioral treatment recommendations, leading providers to feel subjectively pressured to engage patients in medico-centric approaches to care [10]. However, preliminary evidence suggests that MUS patients are seeking alternatives to medical intervention. For example, one study found that patients with MUS are more likely to seek emotional support than a somatic intervention [11].

This lack of concordance between providers and MUS patients about approaches to MUS care may not only contribute to excessive medical treatments, but also poorer treatment outcomes. Illness concordance is defined as a shared understanding between patients and providers about the nature, cause, and best treatment for persistent physical symptoms. Research shows that illness concordance is a strong predictor of treatment outcomes, such as treatment adherence [12], self-management [13], and satisfaction [14, 15]. Unfortunately, there is poor concordance between patients with persistent physical symptoms and providers regarding the nature and treatment of persistent physical symptoms, and particularly among veterans with Gulf War Illness [2]. Veterans with Gulf War Illness are often dissatisfied with care [16], describing encounters with their medical providers as invalidating [17], which contributes to worse treatment outcomes [2].

Developing better patient-provider concordance when treating GWI is inherently difficult because the cause of GWI is unknown and limited medical treatment options render a disease-driven approach ineffective. A person-centered approach to care may be vital in improving concordance and implementing effective treatment. Person-centered health care is defined as care in which “individuals’ values and preferences are elicited and, once expressed, guide all aspects of their health care, supporting their realistic health and life goals [18].” Good person-centered care begins with understanding the whole person, including their life context, values, and importantly the individual’s life and treatment goals. Aligning care with the patient’s values and goals can help providers initiate a concordant treatment plan that improves functioning and quality of life, even in the face of a medical condition that is poorly understood. In other words, identifying patient goals can help providers and patients develop a shared understanding of the best approaches to address the patient’s goals, even though there is limited evidence regarding the cause and physiological underpinning of GWI.

Unfortunately, there is a lack of research on patient-generated goals among veterans with GWI. Studies examining patient goals for those with other persistent physical symptom conditions such as chronic pain and fibromyalgia have found that patients’ goals tend to focus on reducing pain/symptom intensity [19–21], with one study showing that 48% of chronic pain patients rate reducing pain intensity as their top priority [20]. Patients also report improving functioning and quality of life factors, such as physical activity and social relationships, as common treatment goals [22, 23]. However, these studies have two limitations. First, the above studies only examined treatment goals. It is important to understand both treatment and life goals as the latter reflect the “why” behind an individual’s behavior and are an essential component of person-centered care [24]. Research suggests that linking treatment goals to life goals can increase goal attainment and maintenance [25]. Second, the above studies only looked at patient goals in the context of medical encounters and pharmacological treatments, yet clinical practice guidelines encourage providers treating patients with MUS to use non-pharmaceutical, behavioral approaches. To our knowledge, there is only one study to date that specifically examined patient-generated goals within the context of a behavioral therapy among individuals with persistent physical symptoms [26]. Results of this study showed that patients undergoing cognitive-behavioral therapy for chronic pain set goals related to improving quality of life such as increasing physical activity levels, improving functional status and overall wellness, and engaging in recreational activities.

A better understanding of patient-generated goals among veterans with GWI could facilitate a person-centered approach to care. Goals reveal the patient’s targets for change, and can guide practitioners in selecting, tailoring, and implementing treatment options [26]. For example, if a patient with chronic pain has a goal of improving mobility, his provider may suggest a treatment plan that includes education on the pain cycle, pacing activity levels to minimize pain exacerbations, and referral to physical therapy. Such collaborative goal setting is related to increased patient motivation, adherence, and satisfaction with treatment across multiple patient populations and settings [27–30] and has been identified as critical in the treatment of persistent physical symptoms [31, 32].

The current study therefore aims to assess both treatment goals and life goals among veterans with GWI. We did so by asking veterans with GWI about treatment goals and life goals at the start of a randomized clinical treatment trial comparing two behavioral treatments for GWI. Our objective was to identify the most commonly endorsed goals among this population and organize these goals into a typology that can be used to both guide future research and better inform more tailored and efficacious behavioral treatments.

## Materials and methods

The study assessed baseline data collected during a multi-site, randomized clinical trial comparing Problem-Solving Treatment to Health Education among veterans with Gulf War Illness (GWI) [ClinicalTrials.gov Identifier: NCT02161133]. Participants were recruited nationally through letters and flyers as well as various outreach events. Monetary compensation was provided for participation. The study was reviewed by the Veterans Affairs New Jersey Healthcare System Institutional Review Board, Canandaigua VA Medical Center Institutional Review Board, and Edith Nourse Rogers Memorial VA Hospital (now VA Bedford Health Care System) Institutional Review Board (MIRB: 01250). The study was funded by the Veterans Affairs Clinical Sciences Research and Development (VA CSR&D). All participants provided written consent to participate.

### Patient characteristics

The sample included 267 veterans with GWI aged 42–79 years (*M* = 52.89, *SD* = 7.26). The majority identified as male (88%) and white (71.3%). This sample endorsed relatively high levels of symptom severity (M = 14.85, SD = 4.77) as measured by the Patient Health Questionnaire somatic subscale (PHQ-15) [33].

Inclusion criteria included: deployment to Operation Desert Shield/Storm, scores at least half a standard deviation above the mean on the World Health Organization Disability Schedule (WHODAS 2.0) [34], and diagnosis of GWI as measured by the Kansas symptom questionnaire [3]. To meet diagnostic criteria for GWI, veterans needed to endorse moderately severe symptoms in at least 3 out of 6 symptom clusters (fatigue, pain, neurologic/cognitive/mood, gastrointestinal, respiratory, and skin) over at least the past 6 months. Exclusion criteria included: current suicidal or homicidal intent, psychotic symptoms, and/or self-reported diagnosis of a degenerative brain disorder or serious psychiatric or medical illness (e.g., cancer) that could interfere with generalizability of results.

### Materials

As part of a written survey within the baseline assessment, participants were asked two open-ended questions about treatment and life goals. The questions were “What are your goals for treatment?” and “What are your goals for life?” Participants were given the choice to write out their responses and then mail the survey back to study team, or answer these questions over the phone, in which case responses were recorded by a study team member. Multiple responses were allowed, and responses were not restricted in any way. All responses were reviewed to identify common themes and codes.

### Coding procedures

Data were analyzed in multiple phases using Braun and Clarke’s thematic analysis method [35]. In phase one, three coders (LM, ST & NS) reviewed all responses and took notes on initial code ideas. In the second phase, two coders (ST & NS) reviewed all responses again and developed a codebook with a total of 14 individual codes for treatment goals, and 19 individual codes for life goals. These two coders then individually coded the first 25 responses of each question to establish interrater reliability. During this initial coding, inter-rater agreement was 78%, and consensus was achieved in 100% of responses after a brief discussion. The same coders independently coded the remaining responses. Coder consensus was achieved in 100% of responses after discussion. Because we did not limit participant responses, some responses were given multiple codes. In the third phase, two coders (LM & NS) met to review codes for potential themes. In the fourth and fifth phases, codes were consolidated, and specific overarching themes were defined. During this process, codes with low frequency were collapsed with other similar codes. For treatment goals, 1 low-frequency code was collapsed into a larger code, leaving a total of 13 individual codes. LM and NS agreed that the remaining 13 codes fell into 4 overarching thematic categories. For life goals, 4 low-frequency codes were collapsed into larger codes, leaving a total of 15 individual codes. LM and NS agreed that the remaining 15 codes fell into 6 overarching thematic categories.

## Results

### Treatment goals

A total of 262 Veterans (98%) reported their treatment goals at the start of a randomized clinical treatment trial. Five participants’ responses were not coded due to being intentionally skipped or left blank. There was a mean of 1.42 codes per participant. Four overarching thematic categories emerged: 1) Get better/healthier, 2) Improve quality of life, 3) Obtain validation of GWI symptoms, and 4) Don’t know/Don’t have any.

The most prevalent treatment goal theme was to get better/healthier (n = 198, 51.1% of 387 responses, 67.6% of participants). There were four subcategories, or codes, within this theme. These included general goals to get better (e.g., "to be well again”, “I want to get better”; n = 39), as well as specific symptom reduction goals such as improving the physical, emotional, and cognitive symptoms of GWI (e.g., “to get as much relief from the symptoms as possible,” “get rid of my pain,” “improve my energy levels,” “reduce or eliminate the confusion and muddled thinking, and control my anxiety”; n = 87). Additional codes within this theme included prevention of symptom progression/coping with symptoms (e.g., “to prevent memory loss,” “halt the illness”, “learn new coping skills I might not know about”; n = 47), and goals to find and engage in effective treatments for GWI (e.g., “learn about new therapies and treatments for GWI”; “get the best out of my appointments”; n = 25).

Participants also endorsed treatment goals to improve quality of life (n = 112, 28.9% of 387 responses, 34.4% of participants). In contrast to responses within the get better/healthier theme, responses falling within this theme capture goals that are more focused on improving quality of life and functioning beyond physical health or symptoms specific to GWI. Goals grouped in this theme included improving general quality of life (e.g., “I would like to have the best quality of life that can be obtained,” “to gain some kind of quality of life”; n = 31) followed by improving lifestyle factors (e.g., “to have a healthier lifestyle,” “better sleep,” “diet and exercise”; n = 24), and goals to help others (e.g., “pass on knowledge learned from the study to my fellow vets,” “to help out other veterans”; n = 21) and connect with others socially (e.g., “to socialize, I like people,” “spend quality time with my family and friends”; n = 16). Additional quality of life goals included existential/spiritual goals (e.g., “to try to find a purpose in life,” “to be a better person”; n = 15), and financial/vocational goals (e.g., “work and provide for my family”; n = 5).

A slightly smaller portion of participants stated that at least one of their treatment goals was to receive validation for the GWI symptoms (n = 66, 17.1% of 387 responses, 24.8% of participants). There were two subcategories, or codes, within this theme. The first code captured a goal to explicitly receive validation from the government or healthcare providers (e.g., “get the VA to admit that I have it”; n = 5). The second included goals to obtain an explanation for their symptoms (e.g., “figure out why I have symptoms”, “try and understand my GWI”; n = 61). The authors categorized these responses under the validation theme because as a historically contested condition, obtaining an explanation for why GWI developed (e.g., potential exposures to hazardous substances during military service) and understanding the nature of GWI (e.g., symptoms are real, GWI is a real condition) can be extremely validating for veterans with GWI.

Few participants stated that they don’t know/don’t have any specific treatment goals (n = 11, 2.8% of 387 responses, 4.2% of participants).

### Life goals

A total of 263 Veterans (98.5%) reported their life goals. Four participants’ responses were not coded due to being intentionally skipped, unrelated to the question, or otherwise unintelligible. There was a mean of 1.86 codes per participant. The following six overarching thematic categories emerged: 1) Live a fulfilling life, 2) Live a happy life, 3) Live a healthy life, 4) Be productive/financially successful, 5) Manage GWI, and 6) Don’t know/Don’t have any.

The most prevalent life goals were within the theme of living a fulfilling life (n = 188, 37.2% of 505 coded responses, 56.7% of participants). This theme consisted of four main subcategories, or codes. Participants most often reported life goals related to family and social relationships (e.g., “spend more time with family and friends,” “be a good husband and father,” “build healthy relationships and maintain them,” “have a successful marriage”; n = 101). Additional smaller codes included existential goals (e.g., “to live my life to the fullest,” “be the best person I can possibly be”; n = 38), goals related to helping others (e.g., “I want to help others that suffer,” “be an advocate to other women veterans”; n = 31), and spiritual goals (e.g., “live a peaceful life serving God,” “improve my faith in God”; n = 18).

The second largest life goal was to live a happy life (n = 100, 19.8% of 505 responses, 34.6% of participants). There were three subcategories, or codes, within this larger theme which included being/feeling happy (e.g., “just to be happy,” “have peace of mind”; n = 45), enjoying life (e.g., “to enjoy life,” “be stress-free”; n = 32) and engaging in hobbies and enjoyable activities (e.g., “write my book,” “maybe travel a little,” “spend time reading and taking photographs”; n = 23).

Living a happy life was followed closely by a life goal to live a healthy life (n = 99, 19.6% of 505 responses, 32.3% of participants). This theme consisted of three major codes: goals for general good health (e.g., “to live a healthy life,” “to be healthy”; n = 43), goals related to living a long life (e.g., “to live a long life,” “to live to be at least 80 years old”; n = 41), and goals related to living a healthy lifestyle (e.g., “to be active at least four days per week,” “maintain sobriety”; n = 15). Of note, this category did not encompass goals related to specifically improving GWI symptoms, but rather being and living in a healthy manner.

The fourth overarching theme was to live a productive and financially successful life (n = 63, 12.5% of 505 responses, 24.0% of participants). This theme consisted of two codes: career and productivity goals (e.g., “get my degree and take a better job,” “be a productive citizen in society”; n = 35) and goals related to financial success (e.g., “to be financially comfortable to enjoy every day without having to worry about having enough money to make my bills,” “future retirement with financial stability”; n = 28).

A smaller percentage of participants described life goals related to the theme of improving or reversing their GWI (n = 47, 9.3% of 505 responses, 16.3% of participants). Subcategories, or codes, within this theme included learning how to manage GWI generally (e.g., “get effective treatment for my GWI,” “manage my GWI”; n = 12), reducing specific GWI symptoms (e.g., “find a way to live pain-free,” “to manage my headaches and then go from there”; n = 27), and returning to who they were prior to their GWI symptoms (e.g., “feel like I did before the Gulf War”, “get my first life back”; n = 8).

Very few participants stated that they don’t know/don’t have any life goals (n = 8, 1.6% of 505 responses, 3.0% of participants).

## Discussion

This study aimed to identify the commonly reported treatment and life goals for veterans receiving treatment for Gulf War Illness, a highly disabling medically unexplained syndrome. We found that treatment goals among this population fell into the following four themes: 1) Get better/healthier, 2) Improve quality of life, 3) Obtain validation of GWI symptoms, and 4) Don’t know/Don’t have any. We found that life goals among this population fell into the following six themes: 1) Live a fulfilling life, 2) Live a happy life, 3) Live a healthy life, 4) Be productive/financially successful, 5) Manage GWI, and 6) Don’t know/Don’t have any.

Treatment goals were largely focused on getting better/healthier (e.g., improving GWI symptoms, learning to cope with symptoms, preventing symptom progression), with 67.6% of participants endorsing at least one treatment goal to get better/healthier. These findings are largely consistent with previous research examining treatment goals among patients with chronic medical conditions, especially in a treatment-seeking sample such as this one. Similar to our sample, research has shown that treatment goals among individuals with chronic pain, arthritis, and other chronic illnesses are focused on improving physical symptoms such as pain intensity and improving functional status [26, 36, 37].

Life goals among veterans with GWI were predominantly focused on living a fulfilling life. Over half of participants reported at least one life goal related to living a fulfilling life focused on connecting to others, helping others, and finding meaning and spiritual fulfillment. Furthermore, 34.6% of participants reported at least one life goal related to enjoying life. Our findings provide a guide that may be helpful to providers when exploring goals with veterans with GWI. While each veteran’s unique goals should be assessed, these themes could be helpful for providers to keep in mind as potential areas to explore.

It is also important to note that about one fourth of veterans in our sample endorsed treatment goals related to receiving validation of their GWI symptoms. Veterans reported a desire to obtain validation from their providers that their symptoms are real, and to better understand their symptoms. This finding is not surprising considering previous literature that patients with MUS often report feeling dismissed by healthcare providers [38, 39]. However, we also found that 16.3% of veterans in our sample identified at least one life goal related to managing their symptoms of GWI. This is an interesting finding that may be suggestive of the large impact GWI symptoms have had on veteran’s lives. We believe this finding underscores the importance of incorporating validation into the treatment of GWI and building strong patient-provider alliances.

Assessing both treatment and life goals of veterans with GWI is important because doing so may aid in improving patient-provider concordance around GWI management, strengthen veteran-provider relationships, and identify patient-centered treatment objectives. While this study highlights common treatment and life goals for veterans with GWI, future research is needed to establish how to best incorporate both kinds of goals into care. One model that is already widely used within the Veteran’s Health Administration is the VA’s Whole Health Program. In this model, treatment and life goals are assessed, and treatment goals are framed as a means to ultimately achieve life goals. For example, a veteran who is presenting to treatment with a desire to reduce pain may be motivated to engage in treatment to achieve an overarching life goal of being an active and engaged father to his children. Thus, the Whole Health model may be a helpful framework to consider incorporating into the care of veterans with GWI. Our new goal typology can facilitate future research on models of care and allow for cross-study comparison of patient-generated goals among those with GWI.

There are some limitations to the current study. First, the sample was mainly comprised of white, male veterans who use the Veterans Health Administration. Our focus on goals in veterans with GWI addressed a gap in the literature; however, absent other evidence, these results should not be generalized beyond this group. Second, the use of a single question to assess treatment goals and a single question to assess life goals is a limitation. For example, we were unable to assess whether the participants understood the distinction between the questions delineating treatment and life goals or assess any relationship between these two categories of goals.

In sum, this study contributes new knowledge by identifying the common treatment and life goals among veterans with GWI. The findings revealed that veterans with GWI identify treatment goals related to getting better/improving GWI, improving quality of life, and receiving validation of their symptoms, and the large majority of veterans identify life goals related to living a fulfilling, productive, and happy life. Explicitly assessing veterans’ treatment and life goals and working towards a shared treatment goal that incorporates veteran’s overarching life goals may be beneficial to incorporate into routine clinical practice. Doing so could help promote patient-provider concordance around the course of treatment for Gulf War Illness and support a more person-centered approach to care.
