# Correction: Treatment and life goals among veterans with Gulf War illness

**DOI:** 10.1371/journal.pone.0306203

**Published:** 2024-06-21

**Authors:** The PLOS ONE Staff

The following author note is missing from the article: The contents of this article do not represent the views of the U.S. Department of Veterans Affairs or the United States Government.

The publisher apologizes for the error.
